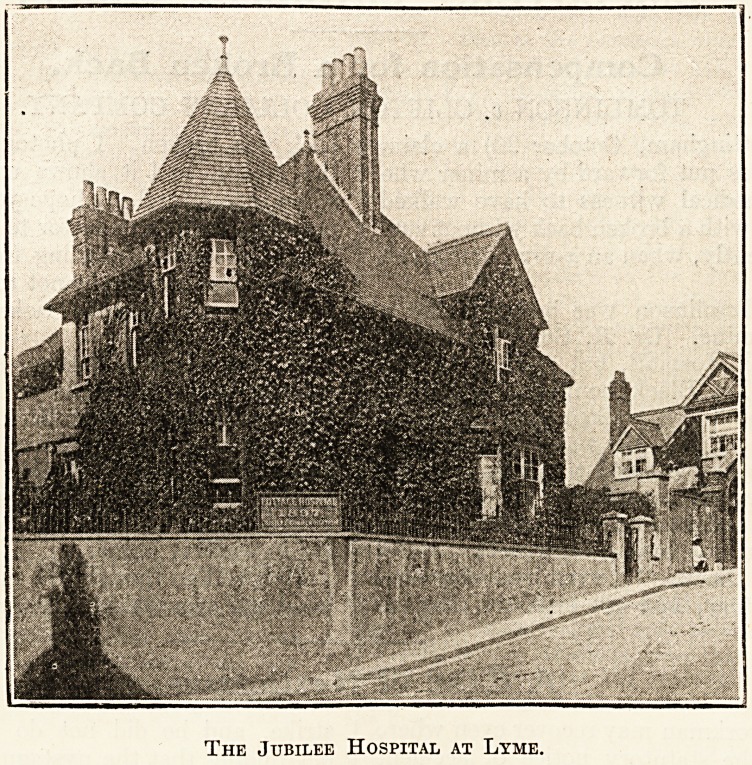# Hospital Architecture and Construction

**Published:** 1911-12-23

**Authors:** 


### HOSPITAL ARCHITECTURE AND CONSTRUCTION.

[Communications on this subject should be marked "Architecture" in the left-hand top corner of the envelope.]

### The Cottage Hospital at Lyme Regis.

This institution is a good example of the transition stage of our small hospitals from their often furtive beginnings to their latest phase as specially-designed, predestinated buildings. In 1873, at a house in Sherborne Lane, the hospital started in a tentative but not ineffective fashion, waiting to impress a sense of its value on the neighbourhood, till the Jubilee of 1887 gave it a chance of obtaining practical recognition for its work. At that time a recently built private house at the corner of Monmouth Street and Church Street fell vacant, and its central situation, combined with its relative size and convenience offered it as the most suitable site available for local hospital work. To it the cottage hospital accordingly was transferred. It should be noted that though the new house had never been designed for hospital purposes, it was capable of being adapted to them, and thus was a true advance on the previous building, which had been chosen at a time when hospital work had yet to begin for the neighbourhood.

The hospital therefore consisted of a male and female ward, with three beds apiece, of a room on the ground floor, used alternately as out-patient department and theatre, of a nurses' sitting-room, bathroom, and of matron's and probationers' bedrooms. The housework here is done mainly by probationers, who are offered one year's training, after which they become cottage nurses in the county. The result of this is that the wages bill is small.

The Jubilee Hospital at Lyme.

Recent alterations in these arrangements have led to various improvements. The medical staff in process of time rebelled against performing operations in a room which was so liable to be infective as an out-patient department, and this now has been exclusively devoted to theatre purposes. It is probable that very few cottage hospitals of the same period, that is adapted merely to their present purpose, possess a theatre on the whole so satisfactory as the one at Lyme Regis. The lighting, it is true, has been a difficulty, but this has been overcome in part by some up-to-date electric lamps, for Lyme is curious not only in its possession of a Corporation, a Cobb, and a fine museum (which after eight years has still to be opened), but also in its possession of electric light. Electricity is also used to warm the theatre. Another improvement has been the setting apart of one of the original probationers' bedrooms for the use of private patients, an arrangement made possible by removing some of the probationers to a house in Monmouth Street which is owned by the hospital, and now joined to it by a covered way.

A glance at the Lyme Regis committee shows that representatives of all classes are to be found upon it. This would seem to imply that the spirit of personal service is a power among the hospital workers of the neighbour-hood, and a comparison of the reports of 1910 and 1911 shows how much this spirit has increased, and how very much the income of the hospital has improved in consequence. In 1911 the annual subscriptions are put down as £181, which is rather more than £50 in excess of those subscribed in 1910.

The latest annual report gives a curious explanation of this: "As regards the funds, we would note that the changed system of collecting which has this year been adopted has amply justified itself by its success. Till the previous year the collecting was undertaken by a paid agent, who received a commission on the amount which he secured. Now the committee undertakes the collecting itself. It would be hard to exaggerate the importance of so momentous a change. The voluntary system cannot, of course, be wholly independent of paid workers, but the task of collecting money can only be persuasive and successful when it is the voluntary work of committeemen and chairman who are known to be doing it for love. The increase in the amount of annual subscriptions is the sign of the results which the voluntary system can attain over any form of paid work for this purpose.

The hospital serves a wide district, including Charmouth on the east, part of Axmouth on the west, together with the inland villages of Uplyme, Wootton, and Whitechurch. Now that the committee is taking in hand the raising of the hospital's income, there is little doubt that these district villages will realise in a way that they have not had an opportunity of doing in the past their relation and responsibility towards the hospital. As, moreover, the district nurse is associated with the hospital and its committee in a very intimate manner, the services of the institution are constantly being brought to the notice of those who receive treatment in their own homes.

The staff recently has experienced several changes. The late honorary secretary, Mr. A. T. M. Bond, has been succeeded in the past eighteen months by the Rev. G. W. Otton, whose secretaryship has been marked, as has been shown, by a vital improvement in organising the hospital's finances. A new matron has also just been appointed in the person of Miss Linford Brown, who, with many Devonshire connections, has come from Honiton, and whose settling down at Lyme has been made somewhat laborious through a shortness of staff due to the illness of one of her probationers.

The new theatre, and above all the increasing activity of the committee, point towards a bright future for this hospital, which has won its way by adapting circumstances, and the work of which on some future occasion will probably demand and receive new and designed quarters of its own.

## Figures and Tables

**Figure f1:**